# Using ethanol as postharvest treatment to increase polyphenols and anthocyanins in wine grape

**DOI:** 10.1016/j.heliyon.2024.e26067

**Published:** 2024-02-08

**Authors:** Modesti Margherita, Alfieri Gianmarco, Magri Anna, Forniti Roberto, Ferri Serena, Petriccione Milena, Taglieri Isabella, Mencarelli Fabio, Bellincontro Andrea

**Affiliations:** a Department for Innovation of Biological, Agrofood and Forest Systems (DIBAF), University of Tuscia, Viterbo, Italy; b CREA - Research Centre for Olive, Fruit and Citrus Crops (CREA-OFA), Caserta, Italy; c Department of Environmental, Biological and Pharmaceutical Sciences and Technologies (DiSTABiF), University of Campania Luigi Vanvitelli, Caserta, Italy; d Department of Agriculture Food and Environment (DAFE), University of Pisa, Pisa, Italy

**Keywords:** *Ethanol postharvest treatments*, *Red wine grapes*, *Polyphenols*, *Anthocyanins*, *Fermentative metabolism*

## Abstract

Red wine grapes are qualitatively evaluated for their content in polyphenols and anthocyanins. Due to certain conditions (weather, latitude, temperature), the concentration of these compounds may be not at the right level for reaching a high-quality wine, thus postharvest technologies can be operated as a remediation strategy. Ethanol is a secondary volatile metabolite and its application has been demonstrated to delay fruit ripening, to reduce decay, and to increase secondary metabolites. The present study investigates the effects of ethanol post-harvest application on wine grapes’ metabolism and composition. Red wine grapes (Vitis Vinifera L. cv Aglianico) were exposed to different ethanol doses (0.25, 0.5, or 1 mL L^−1^) for 12, 24, or 36 h. Ethanol increased sugar concentration, malic acid, free amino nitrogen, polyphenols, and anthocyanins. Particularly, anthocyanins reached an average value of 1820 mg/L in treated samples versus the 1200 mg/L of control grapes already after 12 h whatever the concentration was. Moreover, the highest concentration of ethanol modified berry metabolism shifting from aerobic to anaerobic one. Obtained results suggest that 12 h of ethanol postharvest treatment could be an interesting solution to improve anthocyanins in wine grapes, especially when the quality is not as good as expected.

## Highlights

• Ethanol postharvest treatment increases sugar, malic acid, and free amino nitrogen.
• Ethanol increases polyphenols and anthocyanins in musts after 12 h of treatment.
• 1 mL L^−1^ of ethanol shifts berry metabolism from aerobic to anaerobic ones.

## 1 Introduction

“The French government has announced it is to set aside € 200 m to fund the destruction of surplus wine production in an attempt to support struggling producers and shore up prices and in June, the French agriculture ministry also announced €57 m to fund the pulling up of about 9.500 ha of vines in the Bordeaux region” [1]. The production of ethanol from wine distillation is one of the most used destiny of wine destruction. The European Commission [2] adopted exceptional measures to address the current imbalances in the wine market of several EU regions. Under wine national support programs, it will now be possible for Member States to include crisis distillation to remove the excess wine from the market.

Ethanol is a plant secondary metabolite, which has been declared as a generally recognized safe substance (GRAS) by the Food and Drug Administration (FDA) [3]). Ethanol fumigation has been widely tested on different climacteric and non-climacteric fruits [4]. The major interest for ethanol treatment has been addressed to postharvest pathology, mainly as an alternative to the use of sulfur dioxide (SO_2_) on table grapes. For instance, Lichter et al. [5] showed that dipping grape bunches in 50, 40 or 33% ethanol, before packaging, resulted in inhibition of berry decay equivalent to, or even better than, that achieved by the traditional SO_2_ pre-packaging treatment. Ethanol postharvest treatment can reduce fruits and table grape decay [[5], [6], [7]]). The capacity of ethanol to reduce postharvest decay is mainly related to its aptitude to inhibit respiration and ethylene biosynthesis, therefore reducing metabolic activity, and increasing the storage time [8,9]. As far as wine grapes are concerned, previous research reported that aqueous ethanol applied on vines (Vitis vinifera L. cv Cabernet Sauvignon) increased anthocyanin content in grapes and wines [10]. To date, no studies have been carried out on the use of ethanol as a postharvest treatment on wine grapes. However, the chemical characteristics and biochemical activity of ethanol can suggest a potentially interesting role in modifying quality traits important for wine quality. To begin with chemical traits, ethanol is known to be a powerful solvent capable of extracting different classes of bioactive compounds, such as polyphenols and anthocyanins [11]. When mixed with water, ethanol improves the solubility of bioactive compounds, if compared to pure water, and it has been used as an efficient method to extract polyphenols from grape tissues [12]. The reason behind this seems to be the decrease in solution polarity due to the presence of ethanol which can interfere with the hydrophobic interaction, thus increasing the molecules released in the solution [13]. The increase of bioactive compounds is also associated with ethanol biochemical characteristics. Hence, ethanol has been shown to produce reactive oxygen species (ROS) whose production alters cell membrane properties and permeability, leading to further extraction [14]. Additionally, the presence of ethanol in cells can induce anaerobic conditions and therefore stimulate the activity of fermentative enzymes; these biochemical-induced mechanisms are strongly affected by concentration and treatment time [15]. In view to consume of ethanol from wine sources, a kind of circular economy, we decided, based on previous research on the use of ethanol for postharvest treatment [9,16,17] to study the effect of ethanol postharvest fumigation on wine grape quality. The suggested hypothesis has been that ethanol could i) increase the solubility and the extraction of polyphenols and anthocyanins in grapes and ii) modify postharvest metabolisms by stimulating the activity of different enzymes and therefore changing grapes' composition. Considering that the effect of ethanol on fruit metabolisms varies greatly depending on treatment conditions, different ethanol concentrations and exposition times have been tested.

## 2 Materials and methods

### 2.1 Grape samples and ethanol treatment

Bunches of red wine grapes (*Vitis vinifera* L. cv Aglianico) were carefully hand-harvested in the second half of October in the Avellino area (AV, Italy), and immediately shipped to Tuscia University postharvest laboratory (Viterbo, Italy) in a refrigerated vehicle (at about 10 °C). Grapes were sorted based on the absence of injuries, defects, and fungi infection. The harvested bunches (three sets of 65 kg) were divided each into 13 different lots (about 5 kg each). One lot per sets was used as a time 0 sample and another, without ethanol treatment, as a Control (Ck), for each treatment time. For ethanol treatment, 5 Kg of grapes were placed in 15 L jars (10 L free volume). In each jar, stored a temperature-controlled room (9 m^3^) at 22 ± 1 °C, 2.5, 5.0, or 10 mL of liquid ethanol (99.7% purity. Merck KGaA©, Darmstadt, Germany) was poured on a filter paper in an open Petri dish (to allow the gradual evaporation of ethanol) and immediately sealed. The treatment lasted 12, 24, and 36 h, and at the end of the treatments all the ethanol was evaporated from the filter paper. Each treatment was performed in 3 different jars for a total of 36 jars (plus three time 0).

### 2.2 Color, texture, and chemical analyses

Atmosphere composition inside the jars was measured, at the end of the treatments, by inserting the needle of an Oxycarb infrared analyzer (Isolcell, Bozen, Italy) into the jars before opening them.

10 g of grape juice from the homogenization of 20 berries (20 berries in each jar from different bunches, by three jars) using an Ultraturrax, were centrifuged at 15.000 *g* for 5 min at 4 °C. The obtained supernatant was analyzed by a calibrated Fourier transform infrared Wine Scan FT 120 (Foss Analytics) to determine: total sugars (g L^−1^), pH, titratable acidity (g L^−1^ of tartaric acid equivalent), tartaric acid (g L^−1^), malic acid (g L^−1^), and free amino nitrogen (FAN mg L^−1^). Ethanol content was determined by using the Ethanol Assay Procedure Megazyme (Megazyme Ltd, Bray, Ireland) according to the kit instructions (K-ETOH 08/18). For each parameters and each samples analysis was repeated twice, thus in total six analyses were performed (three jars analyzed twice).

At time 0 and after each treatment, 50 berry samples were immediately analyzed for berry color and skin firmness while other berries were frozen at – 20 °C for further chemical analyses.

The berry skin color was assessed using a Minolta colorimeter (Minolta C2500. Konica Minolta, Ramsey, NY) to determine chromaticity values in terms of CIELab color space, L* (Lightness), a* (green to red), and b* (blue to yellow). For each berry, two reads were taken from the two equatorial faces. The hue angle (h) was calculated by chromaticity values a* and b* using a method reported earlier by McGuire [18]. The berries were then used for measuring the skin resistance by using an Instron Universal Testing Machine (model 3343; Instron Inc., Canton, MA) adapted with a needle tip (1 mm diameter) and performing the test at 10 mm min^−1^ bar speed. Data were expressed as berry skin hardness which was assessed by detecting the maximum break force (F_sk_, N) [19].

### 2.3 Polyphenols and anthocyanins analyses

At time 0 and after each treatment, 20 berries (20 berries in each jar from different bunches, by three jars), were homogenized with 80 % methanol and centrifugated at 4 °C, 10.000 *g* for 15 min. Total polyphenol content was then measured by using the Folin–Ciocalteau method [20], and the obtained results were expressed as mg of gallic acid equivalents (GAE) 100 g fresh weight (FW). On other 20 berries (20 berries each jar from different bunches, by three jars), total anthocyanins were extracted by using pH 3.2 tartaric buffer with 12% ethanol and 1 g/L SO_2_ [21]. Determination of total anthocyanins was performed by the method proposed by Di Stefano and Cravero [21] by appropriate dilution of the samples with a solution consisted of ethanol/water/HCl = 70/30/1. The concentrations of anthocyanins was calculated using the equation: A540 nm (mg/l) = A540 nm*16.7*d where A – absorbance at 540 nm, d – dilution expressed as malvidin-3-glucoside equivalents. The results were expressed as a concentration of malvidin equivalent per L (mg L^−1^). This analysis was repeated twice, thus in total six analyses were performed.

### 2.4 Enzymes activity

#### 2.4.1 Extraction of total soluble proteins for enzymatic assays

For biochemical analyses, one set of berries from different bunches (each set from one jar, thus three sets for each sample) were immediately immersed in liquid nitrogen and ground to a fine powder in a porcelain mortar. 100 mg of powdered grapes tissues was homogenized using potassium phosphate buffer (1:2 w/v) (100 mM, pH 7.5), with thiamine pyrophosphate (TPP; 2 mM), magnesium chloride (2 mM), 2-mercaptoethanol (1 mM) and polyvinyl polypyrrolidone (PVPP; 2% w/v). The homogenate was centrifuged at 15.000 *g* for 20 min at 4 °C and supernatant was used as a crude extract to determine pyruvate dehydrogenase (PDC), alcohol dehydrogenase (ADH), and lactate dehydrogenase (LDH) activities.

The crude extract, for polyphenoloxidase (PPO) activity detection, was obtained as described by Magri and Petriccione [22] with few modifications. The grapes tissue was blended with sodium phosphate buffer (0.5 M, pH 6.4) (1:1.6 w/v) containing PVPP (2% w/v). The mixture was centrifugated at 13.000×*g* for 30 min at 4 °C and the resultant supernatant was collected. For lipoxygenase (LOX) activity, the crude extract was obtained as reported by Ref. [22], with slight modification. Frozen grapes powder was re-suspending with potassium phosphate buffer (50 mM, pH 7.8) (1:3 w/v), containing sodium–EDTA (1 mM, pH 7), and PVPP (2% w/v) and centrifuged at 12.500×*g* for 10 min at 4 °C and the resultant supernatant was collected. Total soluble proteins content in all crude extracts was assayed using the Bradford method [23].

#### 2.4.2 Pyruvate dehydrogenase, alcohol dehydrogenase, and lactate dehydrogenase activities

Pyruvate dehydrogenase activity (PDC) (EC 1.2.1.104) and lactate dehydrogenase (LDH) (E.C. 1.1.1.27) activity were tested by recording nicotinamide adenine dinucleotide (NADH) oxidation at 340 nm for 200 s at 25 °C [24]. PDC mixture assay consisted of 2-(N-morpholino) ethane sulfonic acid (MES; 40 mM; pH 7.5), TPP (1 mM), MgCl2 (1 mM), NADH (0.2 mM), alcohol dehydrogenase (ADH containing 5 U ml^−1^), extract (200 μL), and subsequently sodium-pyruvate (20 mM). The results were expressed as mmol g^−1^ fresh weight (FW). LDH mixture assay was obtained by mixing Tris–HCl buffer (100 mM, pH 7), sodium-pyruvate (20 mM), NADH (0.26 mM), and enzyme extract (100 μL). Reaction mixture was incubated for 50 s before the adding of sodium-pyruvate. The results were expressed as mmol NADH min^−1^ g^−1^ FW.

Alcohol dehydrogenase (ADH) (E.C. 1.1.1.1) activity was measured by monitoring the increase of NADH at 340 nm at 25 °C for 200s [24]. The mixture assay contained KOH-glycine (68 mM, pH 9.0), NAD+ (1.14 mM), ethanol (0.3 M), and enzyme extract (100 μL). Reaction mixture was incubated for 50 s before the adding of enzyme extract. The ADH activity was calculated based on molar absorbance coefficient obtained from NADH (6220 M^−1^cm^−1^). The results were expressed as mmol of NADH generated min^−1^ g^−1^ FW.

#### 2.4.3 Polyphenoloxidase and lipoxygenase activities

Polyphenoloxidase activity (PPO) (EC.1.10.3.1) was assayed as reported by Modesti et al. [25], with slight modifications. Crude enzyme extract (200 μL) was incubated in a catechol solution (0.07 mM catechol dissolved in 200 mM sodium phosphate buffer, pH 6.4) and the absorbance was evaluated at 398 nm for 200 s at 25 °C. The results were expressed as nmol min^−1^ g^−1^ FW. Lipoxygenase activity (LOX) (EC.1.13.11.12) was carried out as reported by Battaglia et al. [26], with some modifications. The assay was conducted in the presence of sodium phosphate buffer (0.1 M, pH 6), linoleic acid sodium salt (5 mM), and crude enzyme extract (25 μL) at 25 °C. LOX activity was evaluated after an incubation of reaction mixture 20 s (without enzyme extract) at 234 nm for 200 s and the results were expressed as mol min^−1^ g^−1^ FW.

### 2.5 Statistical analyses

All data were statistically analyzed through the Shapiro-Wilk and Bartlett test to verify normality and homogeneity of variances. Once these prerequisites were established, collected data were compared by two-way or one-way ANOVA test and Tukey's honestly significant difference (HSD) test with *p* ≤ 0.05 for multiple comparisons. The statistical tests were performed using GraphPad Prism version 3.05 (GraphPad Software, La Jolla, CA, USA). Both chemical and technological data were statistically autoscaled, then employed to carry out a Two-way Cluster Analysis (CA) which was performed, via Ward's method based on principal component analysis (PCA), by using Matlab R2013a (MathWorks®, Natick, MA, USA) and PLS Toolbox (Eigenvector Research, Inc., Manson, WA, USA), for data pre-treatment, and Past ver. 4.11 for Mac [27] for final modeling.

## 3 Results

**Fig. 1:**
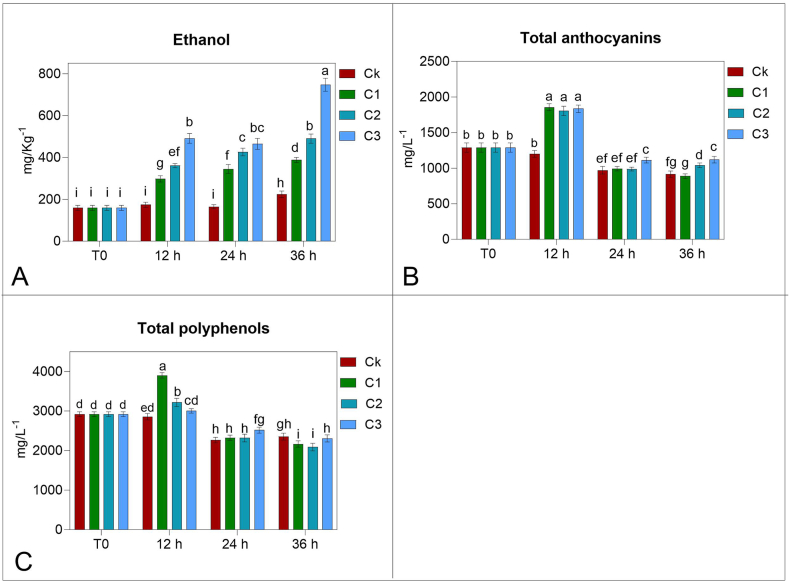
Ethanol, anthocyanins and polyphenols content in grapes after gaseous treatment with 0 (CK), 0.25 (C1), 0.5 (C2) or 1 (C3) mL L^−1^ for 12, 24 or 36 h and at time 0. Data are the mean (±SD) of 6 analyses from 6 different sets of berries sampled from 3 jars, each sample. Different letters within columns indicate statistical significance according to Tukey post hoc test (p < 0.05).

The situation of jar sealing affected the atmosphere around grapes as it is shown in Table 1. After 36 h at 22 °C, in Ck sample, the oxygen concentration was 0, and CO_2_ was very high. In C1 and C2 (the lowest and the intermediate concentration of ethanol), the CO_2_ concentration was lower than in Ck while, at the highest ethanol concentration, CO_2_ content was similar to the one of Ck. Checking the ethanol in the tissue (Fig. 1A), an increased content was observed in Ck at 36 h, even though, as expected, significantly lower l than in the ethanol-treated samples, already after 12 h of treatment. This indicates that ethanol is absorbed rapidly, probably through the stem, as it occurs usually for the gas transport in the berry [28] but, due to its solvent feature, could be absorbed also through the cuticle. The proportion of ethanol content in the tissue of the treated samples reflects the concentration of the treatment, except for the longest treatment time (36 h) where ethanol concentration in the tissue was much higher. This event would highlight an anaerobic respiration taking place intensively.

**Table 1:** Carbon dioxide and oxygen concentration inside the jars after gaseous ethanol treatment with 0 (Ck), 0.25 (C1), 0.5 (C2) or 1 (C3) mL L^−1^ for 12, 24 or 36 h and at time 0. Data are the mean of 1 measurement per jar (3 jars each sample). Different letters within columns indicate statistical significance according to the two-way Anova and Tukey post hoc test (p < 0.05).

Time (h)	Ethanol treatment	CO_2_ mL/Kg hours	O_2_ mL/Kg hours
**12**	**Ck**	10 ± 2 ef	11 ± 2 a
	**C1**	11 ± 1 ef	12 ± 2 a
	**C2**	9 ± 1 f	11 ± 2 ab
	**C3**	11 ± 2 ef	10 ± 2 abc
**24**	**Ck**	16±2c	8±1cd
	**C1**	14 ± 1 cd	10 ± 2 a
	**C2**	13 ± 1 de	9 ± 2 bcd
	**C3**	14 ± 1 cd	7 ± 2 d
**36**	**Ck**	32 ± 3 a	0
	**C1**	24 ± 3 b	0
	**C2**	20 ± 2 b	2 ± 1 e
	**C3**	32 ± 2 a	0

Data are the mean of 1 measurement per jars (3 jars each sample). Value are expressed as mL/Kg of grapes per hours. Different letters within columns indicate statistical significance according to the two-way Anova and Tukey post hoc test (p < 0.05).

Sugar content did not show significant differences after 12 h of ethanol fumigation compared to control and time 0. Increasing the time of treatment (24 and 36 h), the raised sugar content reached the highest values in samples ethanol-treated at 1 mL L^−1^ of concentration (235 and 245 g/L, respectively, for 24 and 36 h) (Table 2). Compared to other samples, malic acid increased in berries treated with 0.25 mL L^−1^ of ethanol, regardless of the treatment time. At higher ethanol concentrations and in untreated sample (Ck), malic acid was lower but higher than the value recorded at the initial time (time 0) (Table 2). Tartaric acid did not show similar behavior, and, at higher concentrations of ethanol, its content seemed to decrease compared to time 0 and control samples (Table 2). Whatever the duration of exposure was, free amino nitrogen (FAN) content significantly increased at higher ethanol concentrations, even though the highest values were observed in grapes treated with 0.5 mL L^−1^ of ethanol. FAN content rose with time only in the Ck sample (Table 2). 12 h of ethanol treatments increased total anthocyanins compared to both Ck and time 0 samples (Fig. 1B). At 24 and 36 h, total anthocyanin content decreased in all samples compared to time 0 and 12 h values. However, in ethanol-treated grapes, a slight but significant increase was observed with respect to control ones, especially when the highest ethanol concentration was applied. In Ck, anthocyanin content decreased significantly in relation to the time of ethanol exposition. Total polyphenol content was affected by the pattern of anthocyanin content: the highest concentration was observed in ethanol-treated samples for 12 h, especially for the lowest ethanol concentration (Fig. 1C). On the other hand, longer treatment times reduced polyphenol concentration compared to samples at time 0. The same behavior was observed in control grapes.

**Table 2:** Sugars, malic and tartaric acids, free amino nitrogen (FAN) content in the berry after gaseous ethanol treatment with 0 (Ck), 0.25 (C1), 0.5 (C2) or 1 (C3) mL L^−1^ for 12, 24 or 36 h and at time 0. Data are the mean (±SD) of 6 analyses from 6 different sets of berries sampled from 3 jars, each sample. Different letters within columns indicate statistical significance according to the two-way Anova and Tukey post hoc test (p < 0.05).

Time (h)	Ethanol treatment	Sugars (g L^−1^)	Malic acid (g L^−1^)	pH	Tartaric acid (g L^−1^)	FAN (mg L^−1^)
**0**		219 ± 8 ef	1.17 ± 0.08 f	3.21 ± 0.02 b	4.34 ± 0.12 b	76.7 ± 6.5 f
**12**	**Ck**	212 ± 10 ef	1.37 ± 0.08 e	3.22 ± 0.01 b	4.45 ± 0.08 ab	91.5 ± 6.2 ef
	**C1**	209 ± 10 f	1.99 ± 0.07 a	3.18 ± 0.01 b	4.31 ± 0.09 b	92.5 ± 7.0 ef
	**C2**	219 ± 10 ef	1.45 ± 0.10 de	3.31 ± 0.02 a	3.62 ± 0.12 e	111.0 ± 6.6 abc
	**C3**	220 ± 9 ef	1.43 ± 0.05 de	3.28 ± 0.02 a	3.56 ± 0.10 e	104.2 ± 9.2 cde
**24**	**Ck**	228 ± 9 de	1.36 ± 0.10 e	3.19 ± 0.01 b	4.59 ± 0.07 a	98.0 ± 6.0 def
	**C1**	230 ± 9 cd	1.71 ± 0.11 b	3.23 ± 0.03 ab	3.73 ± 0.11 e	82.2 ± 5.5 f
	**C2**	230 ± 7 cd	1.14 ± 0.06 f	3.21 ± 0.02 b	4.06 ± 0.11 c	110.4 ± 8.0 abc
	**C3**	235 ± 8 BCE	1.37 ± 0.06 e	3.21 ± 0.02 b	4.17 ± 0.10 cd	110.3 ± 7.9 abc
**36**	**Ck**	219 ± 6 ef	1.39 ± 0.05 e	3.14 ± 0.01 c	4.11 ± 0.06 cd	112.0 ± 8.4 ab
	**C1**	226 ± 8 de	1.62 ± 0.07 bcd	3.22 ± 0.01 b	3.64 ± 0.09 e	102.7 ± 6.6 cde
	**C2**	224 ± 10 de	1.55 ± 0.09 cd	3.32 ± 0.04 a	3.08 ± 0.08 f	117.7 ± 9.7 a
	**C3**	245 ± 10 ab	1.18 ± 0.09 f	3.15 ± 0.03 BCE	3.93 ± 0.09 d	107.8 ± 6.0 bcd

Data are the mean (±SD) of 6 analyses from 6 different sets of berries sampled from 3 jars, each sample. Different letters within columns indicate statistical significance according to the two-way Anova and Tukey post hoc test (p < 0.05).

Concerning the berry skin hardness, the most significant observation is related to a little increase in the applied force for skin breaking observed in ethanol-treated samples compared to the T0 ones (Table 3). Particularly, treated samples at 0.5 mL L^−1^ (C2) showed a significant increase after 24 h, while a recorded but not so significant increase was also observed for 0.25 (C1) and 1.0 mL L^−1^ (C3) after 12 h of treatments. After 12 and 24 h of treatment, control grapes and grapes treated with 0.25 mL L^−1^ of ethanol showed hue value (h°) similar to T0 sample, which practically remained at the same level measured in the T0 sample and in the control (Ck) ones, indifferently from the progressive timing of sampling (12, 24, or 36 h). On the other hand, the h° measured in grapes treated with 0.5 and 1 mL L^−1^ of ethanol appears slightly higher than in samples at T0. Hue angle increased in berries after 36 h of treatment, regardless of the ethanol concentration (Table 3). The difference observed after 36 h was mainly due to the lower colorimetric b* value (data not shown), and it was affected by the colorimetric evolution, into the IV quadrant associated with blue, toward the 360° (or 0°) which is related to purple-red color. Even though no statistical significance has been observed for any differences among data. The same behavior has been observed for L* values which were significantly lower in 36 h samples, regardless of the treatment.

**Table 3:** Berry skin hardness (F_sk_), hue angle, and L* colorimetric attribute of the berry after gaseous treatment with 0 (Ck), 0.25 (C1), 0.5 (C2) or 1 (C3) mL L^−1^ for 12, 24 or 36 h and at time 0. Data are the mean (±SD) of 50 berries from each sample. Different letters within columns indicate statistical significance according to Tukey post hoc test (p < 0.05).

Time (h)	Ethanol treatment	F_sk_ (N)	Hue angle (h°)	Lightness (L*)
**0**		0.188 ± 0.05 d	317.73 ± 50.3	34.83 ± 4.4 a
**12**	**Ck**	0.201 ± 0.05 c	320.90 ± 51.6	33.92 ± 3.7 a
	**C1**	0.205 ± 0.05 BCE	326.71 ± 55.7	32.15 ± 3.9 ab
	**C2**	0.213 ± 0.05 ab	333.31 ± 41.3	29.53 ± 4.5 abcd
	**C3**	0.210 ± 0.05 b	329.94 ± 47	30.71 ± 4.1 abc
**24**	**Ck**	0.205 ± 0.05 BCE	322.24 ± 50.2	29.23 ± 4.8 abcd
	**C1**	0.224 ± 0.05 a	321.90 ± 47.9	26.66 ± 4.9 cd
	**C2**	0.205 ± 0.06 BCE	332.54 ± 45.8	27.44 ± 3.8 cd
	**C3**	0.200 ± 0.05 c	331.98 ± 45.1	28.35 ± 4.1 bcd
**36**	**Ck**	0.202 ± 0.06 BCE	323.24 ± 52.2	23.96 ± 3.7 d
	**C1**	0.183 ± 0.05 d	353 ± 53.3	23.61 ± 3.9 d
	**C2**	0.189 ± 0.06 d	357.83 ± 54.4	23.90 ± 3.9 d
	**C3**	0.204 ± 0.06 BCE	358.21 ± 51.5	26.22 ± 4.6 cd

Data are the mean (±SD) of 50 berries from each sample. Different letters within columns indicate statistical significance according to Tukey post hoc test (p < 0.05).

**Fig. 2:**
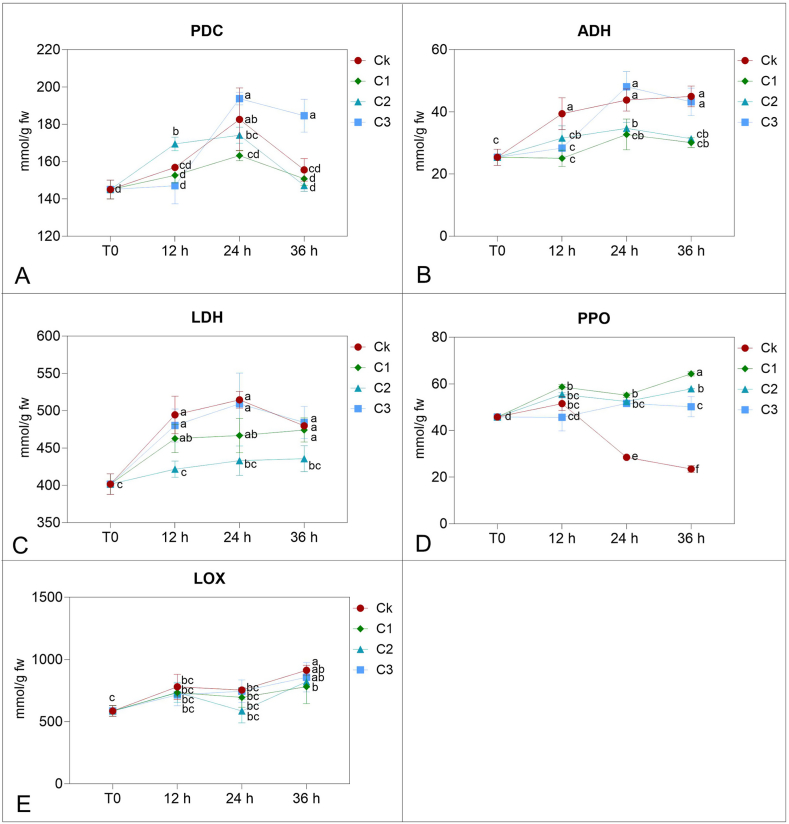
PDC, ADH, LDH, PPO, and LOX activity of the berry after gaseous treatment with 0 (CK), 0.25 (C1), 0.5 (C2) or 1 (C3) mL L^−1^ for 12, 24 or 36 h and at time 0. Data are the mean (±SD) of 3 analyses from 3 different sets of berries, each one from 1 jar (3 jars), each sample. Different letters within columns indicate statistical significance according to Tukey post hoc test (p < 0.05).

After 12 h of treatment, PDC increased significantly in the C2 sample, while a peak after 24 h of treatment was shown in Ck and C3 samples (Fig. 2A). At 36 h, there was a slight not significant reduction of the activity in C3, while a more significant decrease was observed in the other samples. LDH activity increased already at 12 h, then remained in all samples, except than in the C2 sample, being the highest activity detected in Ck and C3 (Fig. 2B). Similarly, ADH had the highest activity in C3 and Ck (Fig. 2C).

PPO activity showed a similar trend in all grapes treated with ethanol with significantly higher value after 36 h in grapes treated with 0.25 mL of ethanol. The Ck sample displayed a decrease in PPO activity after 12 h of treatment until the end of the experiment (Fig. 2D). In LOX activity a slight increase during the experiment was registered, with a significantly higher value in control grapes after 36 h. Ethanol treatment reduced the increase in LOX activity during the whole experiment (Fig. 2E).

**Fig. 3:**
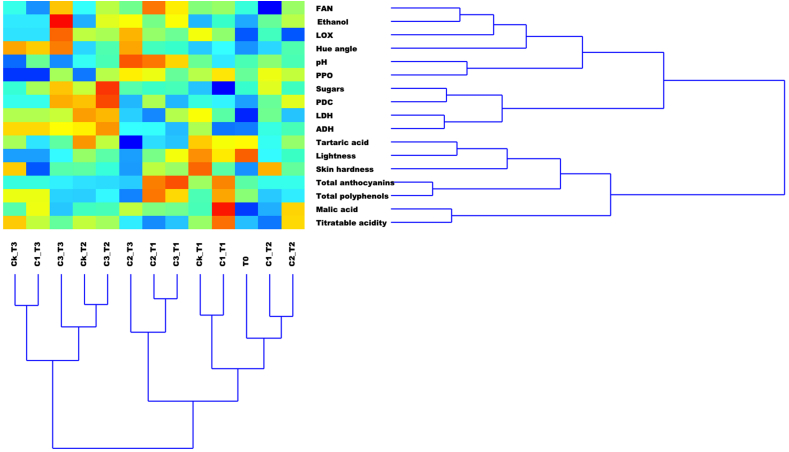
Two-way Cluster Analysis (CA) performed on the data collected on grape berry after gaseous treatment with 0 (Ck), 0.25 (C1), 0.5 (C2), or 1 (C3) mL L^−1^ of ethanol for 12, 24 or 36 h (T1, T2, and T3, respectively) and at time 0 (T0). The two dendrograms graphically represent the combination of scores (left, bottom), and of loadings (right, top), respectively. Indeed, the heat map reports the combination of both factors and the influence on the clustering effect by a coloring based on a scale starting from blue (low) up to red (high).

The two-way cluster analysis (CA) performed on autoscaled data coming from chemical (ethanol concentration, sugars, malic and tartaric acid, FAN, total anthocyanins, total polyphenols, and pH) and technological (Skin hardness, Hue angle, and L*) measurements is reported in Fig. 3, including the two dendrograms referring to score and loading hierarchical segregations, respectively. A heat map, visually showing the influence of the loadings (chemical and technological parameters) on the score clustering is also included.

## 4 Discussion

Ethanol is a plant secondary metabolite that has been shown to accumulate when fruits remain attached to the mother plant for long periods. Ethanol is produced by the ADH enzyme starting from acetaldehyde (AA) under anaerobic conditions, and its biosynthesis is involved in many metabolic/biochemical shifts [29]. In grape berries, during the development phase as well as the overripening stage, a strong overexpression of ADH gene and enzyme activity has been observed [30], therefore a high biosynthesis rate of ethanol has been demonstrated [28]. Thus, the grape cell is used to coexist with a high amount of intracellular ethanol but, when it exceeds a toxic threshold, ethanol is re-oxidized to AA and then to acetic acid, following a detoxification strategy. Ethanol is also known to produce ROS which, in turn, causes oxidative stress inside the cells [31]. The produced ROS can interact with cellular membranes, altering lipids solubility and causing disruption of membrane proteins function [32]. In the last years, several studies have demonstrated that appropriate concentration of ethanol, as postharvest treatment, may have a positive effect on alleviating oxidative stress in papaya [33], fresh-cut burdock [34]and bitter melon [17], reducing malondialdehyde and superoxide anions content. Starting from these assumptions, it is possible to observe that lowest ethanol treatment (C1), at 36 h, looks to show a significant reduction of LOX activity which could contribute to counteract oxidative stress preserving oxidative membrane damage, likely controlling the lipid membrane damage [35].

Exogenous ethanol treatment reduces the PPO activity, slowing down the browning effect, and maintaining the fruit quality after harvest as reported for fresh-cut eggplants [36] and burdocks [34]. Contrarily, in the present study, PPO activity showed higher levels in ethanol-treated samples compared to control. However, it is well known that substrate availability is one of the keys to enzyme activity induction, as well as the optimal pH level, and those effects were recorded even for PPO in grapes[[36], [37]]. As above reported, polyphenol content is observed to increase mainly after 12 h of treatment, particularly and significantly in C1 and C2 treated samples, compared to CK ones. This could have generated an incremented substrate availability for PPO activity which is higher in treated samples than in control ones at 24 and 36 h, also justifying the significant polyphenols reduction observed at 24, and 36 h in treated grapes, nevertheless the potential greater phenol availability due to ethanol effect. A limited induction of PPO activity was also observed in CK at 12 h, but followed by a strong dropdown at 24, and 36 h. The little increase in enzyme activity could partially motivate the reduction in total polyphenols amount observed after 24, and 36 h, also supported by non-enzymatic events of degradation [[38], [39]] which, on the other hands, could also overlap the enzymatic oxidization in ethanol-treated grapes.

Considering the complexity of the metabolic/structural changes induced by ethanol, its postharvest application on fruits can have beneficial or deleterious effects depending on species, concentration, and time of treatment [15]. In the case here reported, the observed sugar increase after 24 and 36 h of treatment, regardless of the ethanol concentration, suggests that a high concentration of ethanol caused its re-oxidation to AA which, in turn, stimulates sugar accumulation, as already served in previous studies [5,33]. Together with sugar, also malic acid increased in samples treated with 0.25 mL L^−1^ of ethanol, especially after 12 h. Similar behavior has been observed in blueberry fruits treated with ethanol [16]. In grape berries, malate becomes available in post-veraison for different catabolism, including the Krebs cycle, respiration, ethanol fermentation, and production of secondary metabolites such as anthocyanins and flavonols [40]. As malate can be used as a substrate for berry fermentation, in the current experiment, ethanol, might have a mass effect inducing ADH activity to oxidize ethanol to AA and then to acetic acid. Acetate will be oxidized in the Krebs cycle, thereby stimulating the Krebs cycle which provokes higher biosynthesis of all involved organic acids, malic acid included. An increase in the Krebs cycle could also induce the synthesis of amino acids and it seems in accordance with FAN content, which increases parallel to the increase of ethanol exposition. At higher concentrations, such as 0.5 and 1 mL L^−1^, the internal content of ethanol seems to be so high that its oxidative and solvent effects could predominate over the biochemical one, favoring the degradation of malic acid after its formation. Finally, ethanol treatments and related ethanol accumulation could be considered as a stressing event, which can stimulate malate degradation addressed to the gluconeogenesis event [41]. This supposition seems to be in part confirmed by the observed increase in sugar concentration.

A general, even slight, effect of the ethanol application in enhancing a firmness response of the tissue would be in line with what just recently [42] or previously [5]observed by other authors in table grapes, or other fruits [16].

Concerning the colorimetric evaluation, as described before, the computed hue angle (h°) deriving from the CIELab coordinates a* and b* shows a quite clear displacement from the blue color of the T0 sample and the control ones (regardless of the sampling time) to the purple-red of the grape ethanol-treated for 36 h, regardless of the alcohol concentration. Nevertheless, due to very high standard deviations, the observed differences did not result significantly. The specific effect of the ethanol in terms of anthocyanin enhancement is discussed below but, about the color manifestation, a possible methylation effect on specific anthocyanins, probably due to a stressing even associated with ethanol action, could be suggested. As already known, the reduction of the ratio between hydroxylates and methoxylated anthocyanins can be considered one cause of the grape color modification toward the red tonalities [43,44]. In any case, an enhancement of the grape peel color due to spray ethanol treatments has been already observed in previous experiments, even carried out in pre-harvest of red wine grapes [45].

As far as concerns the lightness (L*), its progressive reduction, associated with the sampling time more than to the effect of ethanol treatments (as it is observed also in control grapes), is parallel to a slight, but anyway, present pH lowering. Heredia et al. [413have observed and discussed the pH action in affecting the grape berry lightness, and by underlying the L* increment coupled to a pH rose.

The observed reduction of tartaric acid at higher ethanol concentrations and longer treatment time, strengthen the hypothesis of oxidative effect induced by ethanol; hence, in wine and grapes, when tartaric acid is oxidized, it forms glyoxylic acid and xanthylium salt which leads to color changes [46]. The increase of anthocyanins observed after 12 h of treatments, with progressive significance at 0.25 and 0.5 mL L^−1^, clearly displayed in the CA even in combination with the total polyphenol influence, can be drawn back to two hypotheses. On one hand, it is known the ethanol effect in increasing the polyphenol solubility, including the larger and more hydrophobic phenolics. Hence, ethanol facilitates the release of anthocyanins from the skin thanks to the decrease of solution polarity, and disruption of hydrophobic interaction, therefore increasing the release of molecules in the solution [13]. Additionally, Medina-Plaza et al. [47], stated that the increase of ethanol concentration increases the association of anthocyanin with molecules in solution rather than binding sites on the cell wall materials. Another hypothesis is suggested by other studies, where is reported that at a certain concentration, ethanol favors the anthocyanin biosynthesis in berry tissues [10]. Indeed, it has been suggested that the UDP-glucose: flavonoid 3-*O*-glucosyltransferase (UFGT) gene, which catalyzes the final step in anthocyanin biosynthesis, is activated [10]. However, we cannot exclude that the other genes involved in anthocyanin biosynthesis could be stimulated as well.

In the present study the influence of ethanol treatment on its metabolism in grapes has been investigated, by analyzing PDC, ADH, and LDH. PDC and ADH catalyze the synthesis of acetaldehyde from pyruvate and ethanol from acetaldehyde, respectively. The high activities of PDC and ADH suggest an increase of acetaldehyde and ethanol in grapes during 36 h of treatment as reported by Toro and Pinto [48]. Furthermore, ADH activity is affected by ethanol concentration and this enzyme is a key biochemical step in fruit responses to hypoxic conditions. Several studies have demonstrated the increase of PDC and ADH activities with the occurrence of anaerobic respiration during postharvest in different fruit crops [49,50]. After 12 h of treatment, PDC increased significantly in the C2 sample, while a peak after 24 h of treatment was shown by Ck and C3 (the highest content of ethanol) samples, meaning that this enzyme was working significantly in transforming pyruvic acid to lactic acid or acetaldehyde. At 36 h, there was a slight not significant reduction of the activity in C3, probably due to the continuous anaerobic activity, while a more significant decrease was observed in the other samples, inversely linked to the ethanol concentration measured in the tissue ethanol-treated. LDH activity increased already at 12 h, then still remain in all samples, except than in C2 sample, being the highest activity detected in Ck and C3. ADH (ethanol to acetaldehyde, thus detoxifying ethanol) confirmed this behaviour: C3 and Ck had the highest activity notwithstanding ethanol concentration in the tissue was very different. An observed curious response: the sample presenting the highest ethanol concentration (C3 at 36 h) and the untreated one (Ck) showed very similar anaerobic enzymes activities. Thus, it can be assumed that the lowest ethanol concentration (C1 and overall C2) inhibited respiration, aerobic and anaerobic, as it has been observed in other fruits [17,33,34], while at the highest concentration, there was a stressing effect stimulating anaerobic respiration. It is presumed that, after 12 h, the atmosphere concentration still permitted aerobic respiration but, successively, when a significant atmosphere modification is observed overall in Ck and C3, it affected the respiration. This last observation is also shown by its last atmosphere concentration recorded, where CO_2_ increases and oxygen decreases much more compared to the first two samplings highlighting an anaerobic respiration taking over. Thus, it is possible to conclude that the effect on the anaerobic enzymes such as PDC, LDH, and ADH, is due more to gas concentration than ethanol accumulation in the tissue. This response is obvious because the cell metabolism is used to be affected by oxygen or carbon dioxide, and not by ethanol thus the receptors are more sensitive to their variations, and to survive, plants utilize different mechanisms to respond and adapt to continuously changing environmental factors [51,52].

In hierarchical representation, a clustering effect among T0 samples, control ones at 12 h (Ck_T1), and treated grape samples at 12 (T1), and 24 h (T2), particularly for low and medium ethanol concentrations (C1 = 0.25, and C2 = 0.51 mL L^−1^) is quite well defined. In terms of loading effect, the T0 score is associated with skin lightness, which slightly influences even the Ck_T1 one, while the C1_T1 score is significantly marked by malic acid and, at descendent impact, by titratable acidity, total anthocyanins, and polyphenols. The influence of these two last loadings also appears on C2_T1 and C3_T1 scores, confirming the observed action of the ethanol treatments, after 12 h and regardless of the concentration, in enhancing those metabolites. A second distinct cluster looks separated from the first just described. Grape samples at the highest ethanol concentration of treatment and after 24 h (C3_T2) are significantly affected by sugars and PDC activity and, with a lower influence, by ADH and LDH enzymes. This score is associated with that derived from the same ethanol treatment at the highest time of exposition (C3_T3), which is, in turn, affected by the strongest ethanol accumulation, and by an evident LOX and hue angle influence, while a less marked but present action is due to FAN. These last observations seem to confirm that prolonged ethanol exposition has a role in promoting a postharvest ripening process, parallel to the shifting of the intracellular metabolism towards a fermentative action which has been discussed before.

Practically speaking, with about 10–20 L of liquid ethanol (99%) in a fog producer device, it is possible to reach the required ethanol concentration in an 85 m^3^ room, which is loadable with about 4.5 tons of grape. The treatment can be performed at room temperature. Considering about 20 € per L of ethanol food grade, the cost of ethanol will be 200–400 €, namely 8 cents per kg of grape using 20 L of ethanol food grade. However, it is important to highlight some important limitations. Firstly, the study primarily focuses on specific time points and ethanol concentrations, and a more comprehensive investigation covering a broader range of exposure durations and concentrations could provide a more nuanced understanding of the dynamics involved. Additionally, the complex metabolic responses induced by ethanol are influenced by various factors, including grape varieties, storage and environmental conditions, which were not explored in this study. Future research incorporating a more diverse set of parameters and conditions would enhance the generalizability of the findings. Furthermore, the interpretation of results is constrained by the inherent variability in biological systems, and caution should be exercised in extrapolating the findings to different grape varieties or environmental settings. Despite these limitations, our study contributes valuable insights into the biochemical responses of grapes to ethanol treatments under the specific conditions examined.

## 5 Conclusions

Postharvest short-time treatment (12 h) of red wine grapes with gaseous ethanol application could represent an interesting procedure aimed at enhancing the wine quality which offers interesting perspectives for improving berry color and bioactivity when wine grapes reach the harvest with anthocyanin deficiency, a phenomena which is strictly related to the climate modifications and weather consequences. Ethanol, a simple organic compound, quite easily useable for postharvest application on wine grape berries could be a sustainable approach in this sense, especially right now where an excess of wine requires its distillation to ethanol. Among the three concentrations (0.25, 0.5, and 1 mL L^−1^) as well as the three times of exposure (12, 24, and 36 h), the shortest time treatment (12 h) allowed to increase anthocyanins (about 40%), whatever the ethanol concentration was. As the main response of the ethanol treatments, an increase in sugar, malic acid, and free amino nitrogen (FAN) concentration was also observed. On the other hand, under the effect of the strongest and more prolonged ethanol action, an intracellular anaerobic metabolism was induced, with the involvement of several key enzymes (e.g. ADH, PDC, and LDH). Pattern recognition of the data performed by a multivariate approach allowed us to obtain a concomitant definition of the influence of chemical and technological variables in discriminating the grape samples based on the timing and/or the concentration of ethanol exposition. The sensitivity of wine grapes to various environmental factors underscores the importance of strategies that can positively impact their metabolism and composition. As the present study demonstrates, ethanol treatment emerges as a promising avenue in this regard, offering potential benefits in cases where polyphenol and anthocyanin concentrations may fall short of the desired levels for producing high-quality wine. In conclusion, the findings of this study not only deepen the understanding of the intricate metabolic responses of grapes to ethanol treatments but also hold significant implications for the postharvest management of grapes quality. These insights pave the way for informed strategies that harness ethanol's potential to modulate metabolic pathways, offering practical applications in enhancing the technical quality of wine grapes during postharvest handling.

## Funding

This research did not receive any specific grant from funding agencies in the public, commercial, or not-for-profit sectors.

## CRediT authorship contribution statement

**Modesti Margherita:** Writing – review & editing, Writing – original draft, Methodology, Investigation, Formal analysis, Data curation. **Alfieri Gianmarco:** Writing – review & editing, Formal analysis. **Magri Anna:** Methodology, Formal analysis, Data curation. **Forniti Roberto:** Writing – review & editing, Methodology, Formal analysis. **Ferri Serena:** Methodology, Formal analysis, Data curation. **Petriccione Milena:** Writing – review & editing, Writing – original draft, Supervision, Investigation, Formal analysis, Data curation. **Taglieri Isabella:** Writing – review & editing, Formal analysis. **Mencarelli Fabio:** Writing – review & editing, Writing – original draft, Supervision, Investigation, Data curation, Conceptualization. **Bellincontro Andrea:** Writing – review & editing, Writing – original draft, Supervision, Investigation, Data curation, Conceptualization.

## Declaration of competing interest

The authors declare that they have no known competing financial interests or personal relationships that could have appeared to influence the work reported in this paper.
